# Genomic Insights into and Lytic Potential of Native Bacteriophages M8-2 and M8-3 Against Clinically Relevant Multidrug-Resistant *Pseudomonas aeruginosa*

**DOI:** 10.3390/antibiotics14020110

**Published:** 2025-01-21

**Authors:** Francisco Ricardo Rodríguez-Recio, Javier Alberto Garza-Cervantes, Francisco de Jesús Balderas-Cisneros, José Rubén Morones-Ramírez

**Affiliations:** 1 Facultad de Ciencias Químicas, Universidad Autónoma de Nuevo León (UANL), San Nicolás de los Garza 66455, Mexico; francisco.rodriguezrc@uanl.edu.mx (F.R.R.-R.); javier.garzacn@uanl.edu.mx (J.A.G.-C.); francisco.balderascs@uanl.edu.mx (F.d.J.B.-C.); 2 Centro de Investigación en Biotecnología y Nanotecnología, Facultad de Ciencias Químicas, Universidad Autónoma de Nuevo León, Parque de Investigación e Innovación Tecnológica, Apodaca 66628, Mexico

**Keywords:** multidrug-resistant bacteria, *Pseudomonas aeruginosa*, bacteriophage therapy, antibiotic resistance, environmental phages

## Abstract

**Background/Objectives:** Antibiotic resistance in pathogenic bacteria poses a critical global health threat, with multidrug-resistant (MDR) strains increasingly undermining conventional treatments. Among these, *Pseudomonas aeruginosa* is a high-priority pathogen due to its resistance to carbapenems and frequent presence in hospital settings, contributing to severe healthcare-associated infections. This study aimed to isolate and characterize novel bacteriophages from environmental wastewater samples that could specifically target MDR *P. aeruginosa*. **Methods:** Two bacteriophages, M8-2 and M8-3, were isolated from wastewater in Monterrey, Mexico. A genomic analysis classified M8-2 and M8-3 within the *Caudoviridae* family, and next-generation sequencing (NGS) was used to confirm the absence of undesirable antibiotic resistance or virulence genes. Optimization of viral amplification was performed to achieve high titers, with structural proteins characterized by SDS-PAGE. **Results:** Phages M8-2 and M8-3 exhibited specific lytic activity against MDR strains of *P. aeruginosa*, offering a targeted approach to combat antibiotic-resistant infections. High genetic similarity (>95%) to known Gram-negative bacterial phages was observed. Optimized viral amplification yielded titers of 4.2 × 10^7^ and 1.03 × 10^9^ PFUs/mL for M8-2 and M8-3, respectively. The specificity of these phages minimized disruption to the host microbiome, and their significant efficacy in suppressing bacterial growth positions bacteriophages as promising candidates for localized and personalized phage therapy, especially in chronic and hospital-acquired infection settings. **Conclusions:** These findings highlight the therapeutic potential of M8-2 and M8-3 in addressing antibiotic-resistant *P. aeruginosa* infections. Their safety profile, high target specificity, and robust lytic activity underscore the feasibility of incorporating phage-based strategies into current antimicrobial protocols. This study contributes to the broader goal of developing sustainable and effective phage therapies for diverse clinical and environmental contexts.

## 1. Introduction

Bacterial infections continue to be a major global health concern, with the development of antibiotics representing a transformative advancement in medicine that fundamentally changed the management of infectious diseases. However, the rapid adaptability of bacteria has driven the emergence of diverse antibiotic resistance mechanisms, giving rise to multidrug-resistant bacteria (MDRB). MDRB now pose an increasingly significant threat to public health, leading to an estimated 700,000 deaths annually—a figure projected to rise to 10 million by 2050 if effective, new treatments are not developed [1,2].

Among MDRB, *Pseudomonas aeruginosa* is a critical-priority pathogen due to both its resistance to carbapenems, one of the last-resort antibiotics, and its high prevalence in hospital environments. This combination increases the risks of hospital-acquired infections, contributing to higher rates of readmission, extended morbidity, and greater mortality [3,4]. *P. aeruginosa* is associated with a variety of serious clinical conditions, particularly in immunocompromised patients and those with chronic illnesses. Notable examples include hospital-acquired pneumonia—such as ventilator-associated pneumonia—and lung infections in individuals with cystic fibrosis, where colonization can lead to chronic, life-threatening disease. Additionally, it frequently causes severe burn wound infections, bloodstream infections (bacteremia), and urinary tract infections, especially in clinical settings. Collectively, these infections are notorious for their high mortality rates, increased healthcare costs, and significant public health burden, further underscoring the need for innovative antimicrobial solutions against this pathogen. The persistence of *P. aeruginosa* and similar MDRB in healthcare settings highlights the urgent need for alternative, targeted therapeutic approaches. Unlike broad-spectrum antibiotics, which often disrupt the complex microbial communities within the human body and increase susceptibility to subsequent infections, these new approaches should minimize collateral damage to the beneficial microbiota [5]. For the purposes of this study, we refer to bacteria as “multidrug-resistant” when they exhibit nonsusceptibility to at least one agent in three or more antimicrobial classes, consistent with definitions used by the European Centre for Disease Prevention and Control (ECDC) and the Centers for Disease Control and Prevention (CDC).

Traditional antimicrobial agents act by interfering with cellular processes such as cell wall synthesis, membrane integrity, or enzymatic functions critical for microbial growth or survival. However, antimicrobial resistance (AMR) occurs as microorganisms acquire mutations or gene transfers that allow them to evade these mechanisms. This resistance is driven in large part by the overuse and misuse of antimicrobials in both medical and agricultural settings, accelerating the development of resistant strains and rendering many standard treatments ineffective [6].

The World Health Organization (WHO) recognizes AMR as a critical global health threat, urging the development of novel therapeutic agents with diverse chemical structures and innovative mechanisms of action to combat this growing crisis. The challenge is especially acute with MDRB such as *P. aeruginosa*, which shows high adaptability and resistance to conventional treatment options [7].

Numerous studies have demonstrated the potential of phages against *P. aeruginosa* in both laboratory and clinical contexts. For instance, lytic phages targeting *P. aeruginosa* have been shown to reduce bacterial loads in animal models of burn wound infections (C. S. McVay, M. Velásquez and J. A. Fralick) [8], attenuate lung infections, and even mitigate biofilm formation (M. Rossitto, E. V. Fiscarelli and P. Rosati) [9]—A critical aspect of pathogen resilience. Engineered or natural phage cocktails have also illustrated how broad-spectrum approaches can curb the emergence of phage-resistant bacterial mutants (F. Forti, D. R. Roach, M. Cafora, M. E. Pasini, D. S. Horner, E. V. Fiscarelli, et al.) [10], further supporting the feasibility of phage therapy as an adjunct or alternative to standard antibiotic regimens. Collectively, these efforts underscore the need to explore locally sourced phages like M8-2 and M8-3, which may offer enhanced effectiveness against regional clinically relevant strains of *P. aeruginosa*.

In this context, bacteriophages (phages)—viruses that naturally prey on bacteria—provide a promising alternative due to their high specificity and potential to target resistant bacterial populations. Representing the most abundant biological entities on Earth, phages co-evolve with bacterial hosts, allowing them to effectively control specific bacterial populations without affecting human cells or the beneficial microbiota. Phage therapy (PT) harnesses this specificity, offering targeted, effective treatment against bacterial infections, including those caused by MDRB such as *P. aeruginosa* and *Staphylococcus aureus* [11,12,13,14]. In addition to their narrow host range, which reduces collateral harm to commensal microorganisms, phages can be employed alongside traditional antibiotics, potentially enhancing therapeutic outcomes through synergistic effects. Their ability to self-amplify at the site of infection ensures a localized and sustained antibacterial effect, while their adaptability to evolving bacterial resistance mechanisms further distinguishes them from conventional therapies. These attributes collectively highlight the advantages of bacteriophages as a research approach for novel anti-infective strategies, particularly in the fight against multidrug-resistant pathogens. Unlike antibiotics, PT targets only pathogenic populations, preserving the host’s microbiome and reducing the risk of off-target effects—a critical advantage for personalized medicine.

Phage therapy has demonstrated efficacy that is comparable to, and in some cases surpassing, conventional antibiotic treatments in preclinical and clinical studies [15]. Beyond their direct antibacterial actions, phages can synergize with the human immune system, particularly the complement system, to enhance bacterial clearance. Additionally, their abilities to proliferate and disseminate systemically enable phages to provide ongoing protection against recurrent infections [16,17,18,19].

This study emphasizes the therapeutic potential of environmental phages, particularly those native to the Monterrey Metropolitan Area (MMA), for treating MDRB. Phages isolated from local environments may offer heightened effectiveness against indigenous bacterial strains due to shared ecological pressures. Here, we report the isolation and characterization of two bacteriophages specific to multidrug-resistant *P. aeruginosa* strains from MMA. These phages provide a foundation for localized phage therapy, offering a targeted antimicrobial approach that minimizes disruption to the host microbiome. In light of the pressing need for innovative antimicrobial strategies, this work contributes to the broader goal of identifying and developing novel agents to combat AMR with precision and sustainability.

## 2. Results

### 2.1. Isolation and Initial Confirmation of Bacteriophages Using a Double-Layer Agar (DLA) Assay

**Figure 1:**
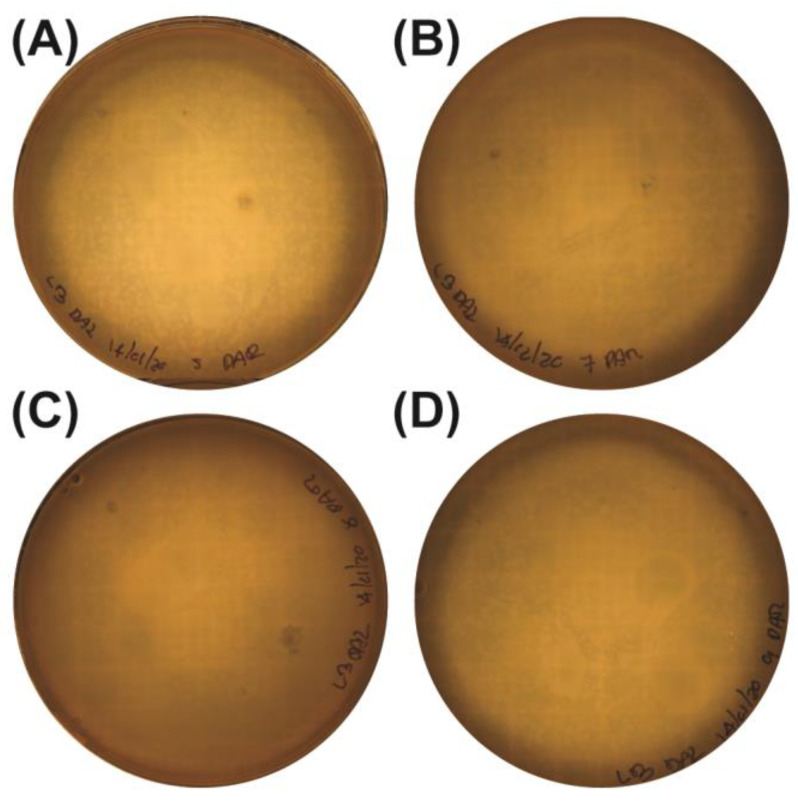
Double-layer agar assay (DLA) showing lytic zones in the multidrug double-layer agar (DLA) method adapted from Adams for *P. aeruginosa* . The representative samples from the Topo Chico stream are illustrated: (**A**) Sample 5, (**B**) Sample 7, (**C**) Sample 8, and (**D**) Sample 9.

To detect and isolate bacteriophages capable of lysing multidrug-resistant *P. aeruginosa*, DLA assays were performed on environmental water samples collected from the Topo Chico stream. Using the multidrug-resistant *P. aeruginosa* strain as the bacterial host, zones of lysis were observed on the bacterial lawns, indicating the presence of phages specific to this host. In total, eleven plates showed turbid lytic plaques, suggesting phage activity against the *P. aeruginosa* strain (Figure 1). These initial observations supported further investigation into the potential of these phages as therapeutic agents targeting antibiotic-resistant pathogens.

### 2.2. Verification of Phage Specificity Using a Spot Test Assay

**Figure 2:**
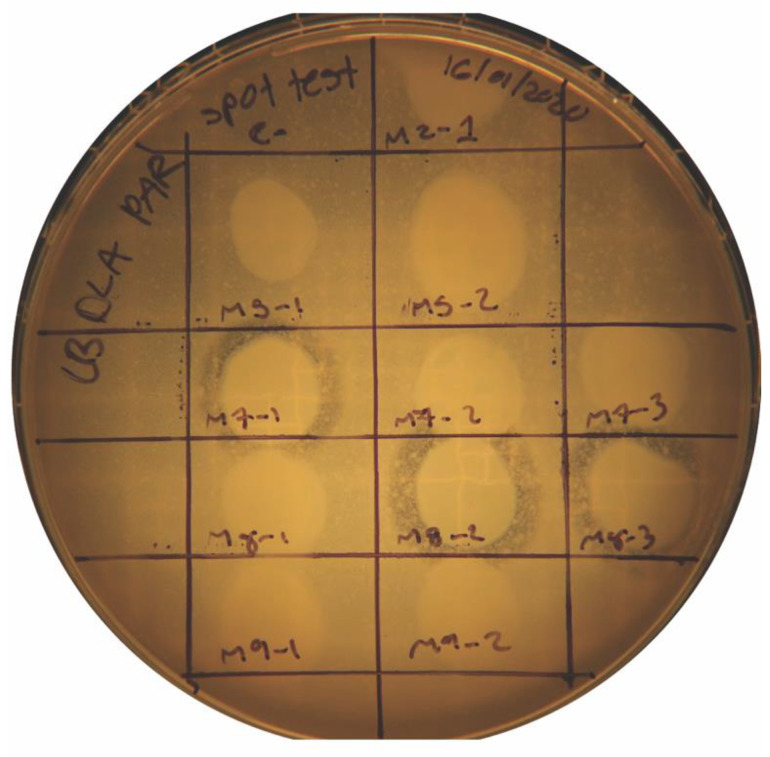
Spot test assay of multidrug-resistant *P. aeruginosa*. Lytic activity is evident in samples M7-1, M8-2, and M8-3, confirming the presence of phages. Lysogenic contamination and turbid lytic zones are also visible.

To confirm the viral origin of the turbid zones observed in the DLA assay, a spot test was conducted. This method validated the isolation of three phages—designated M7-1, M8-2, and M8-3—which demonstrated distinct lytic activity against the multidrug-resistant *P. aeruginosa* strain (Figure 2). The presence of clear inhibition zones on the bacterial lawn further confirmed phage specificity. However, in addition to the target lysis, turbid zones containing lysogens and some cross-contamination with the *P. aeruginosa* host strain were observed, indicating potential temperate phage behavior in some samples.

### 2.3. Quantification and Titration of Bacteriophage Stocks

**Figure 3:**
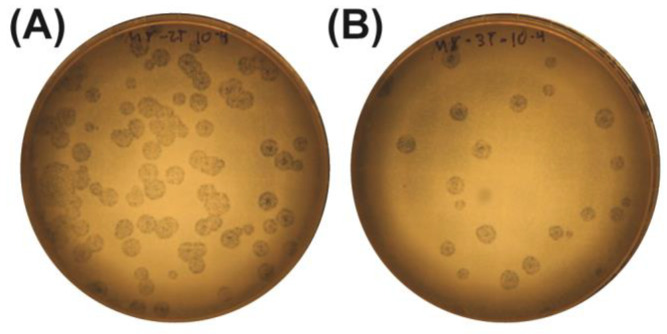
Serial dilution assays for the viral titer determination. (**A**) Phage M8-2 and (**B**) phage M8-3. Inhibition zones with diameters <1 mm can be observed within the plaques, indicating efficient phage lysis.

Phage stocks M7-1, M8-2, and M8-3 were further quantified through serial dilutions on DLA to establish viral titers. This procedure not only allowed for an accurate viral quantification but also facilitated the isolation of homogeneous lytic plaques from each sample. Plaques generated by dilutions at a 10^−4^ factor for M8-2 (Figure 3A) and M8-3 (Figure 3B) were selected for plaque-forming unit (PFU) counting. The calculated viral titers are presented in Table 1.

### 2.4. Amplification of Bacteriophages Using the Lysed Plate Assay

**Figure 4:**
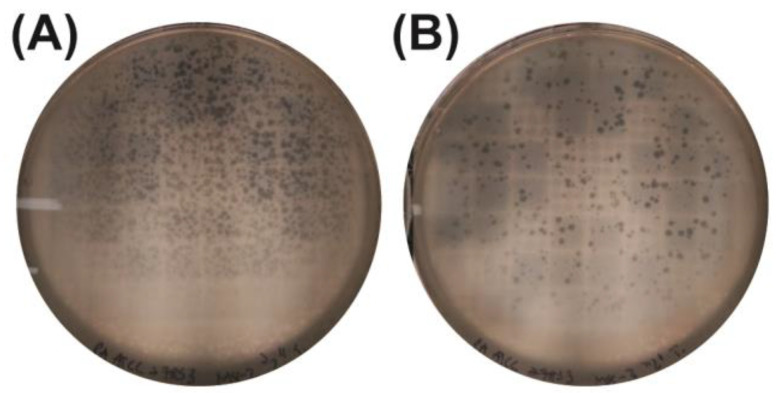
Amplified bacteriophage populations in *P. aeruginosa* culture. (**A**) Phage M8-2 and (**B**) phage M8-3 show fully lytic non-confluent plaques, indicating successful amplification.

**Figure 5:**
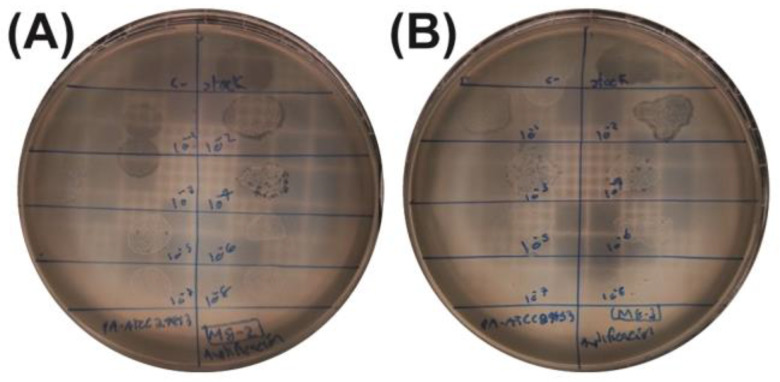
Viral titration following lysed plate amplification. (**A**) Phage M8-2 and (**B**) phage M8-3.

To increase phage yields and establish a clonal phage population, amplification was performed via a lysed plate assay with the multidrug-resistant *P. aeruginosa* host. High-density lytic plaques formed on the plates (Figure 4), allowing for phage recovery from the soft agar. After filtration, viral titers were quantified (Figure 5 and Table 2). Following this amplification, phages M8-2 and M8-3 achieved titers of 4.2 × 10^7^ PFUs/mL and 3.0 × 10^7^ PFUs/mL, respectively. These titers reflect the substantial yield and complete lytic capacity of the amplified phage populations, as evidenced by the extensive inhibition zones observed in the DLA assays.

### 2.5. Optimization of the Viral Yield Through a Determination of the Optimal Dilution via the Lysed Plate Assay

To maximize the viral yield, titration tests were conducted on bacteriophage stocks M8-2 and M8-3 using the double-layer agar (DLA) method to establish optimal dilution factors. The approach involved recovering all phages present in soft agar across a range of dilution factors, followed by re-titration to facilitate rational amplification. This process aimed to identify the phage concentration that effectively lyses a substantial portion of the bacterial host population without achieving complete eradication, ensuring that each phage particle has the opportunity to infect and replicate within a bacterial cell.

**Figure 6:**
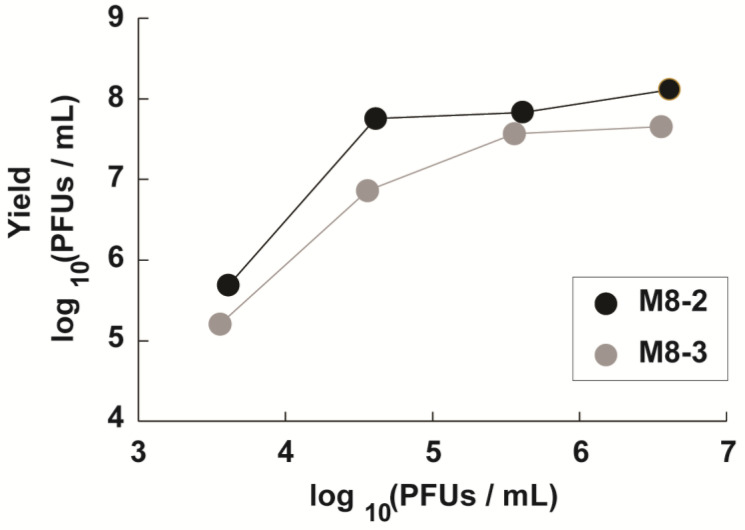
Optimal dilution determination for maximizing the viral yields of phages M8-2 and M8-3. Error bars indicate standard deviations. All experiments, including controls, were conducted in triplicate, with significant differences highlighted by Tukey’s test (α = 0.05).

The results for phages M8-2 and M8-3 are presented in Figure 6. For phage M8-2, which was recovered at a 10^1^ dilution factor, an initial concentration of 4.1 × 10^6^ PFUs/mL (Log_10_ = 6.61) yielded a maximum output of 1.3 × 10^8^ PFUs/mL (Log_10_ = 8.11). The statistical analysis using Tukey’s test (α = 0.05) confirmed a significant difference in the viral yield when employing this high viral concentration compared to lower concentrations (≤4.1 × 10^4^ PFUs/mL, Log_10_ = 3.61), validating the efficacy of the selected dilution for optimal phage amplification.

Similarly, phage M8-3, which was also recovered at a 10¹ dilution factor with an initial concentration of 3.6 × 10^6^ PFUs/mL (Log_10_ = 6.56), achieved a peak yield of 4.5 × 10^7^ PFUs/mL (Log_10_ = 7.65). Tukey’s test further confirmed a statistically significant increase in yield at this higher viral concentration in comparison to lower concentrations (≤3.6 × 10^4^ PFUs/mL, Log_10_ = 3.65), indicating that high initial viral loads are critical for optimizing phage production. This optimized dilution protocol offers a reproducible approach for achieving high viral yields, which are essential for subsequent applications and therapeutic evaluations of phages M8-2 and M8-3.

### 2.6. Co-Culture Assays of Phages with P. aeruginosa

**Figure 7:**
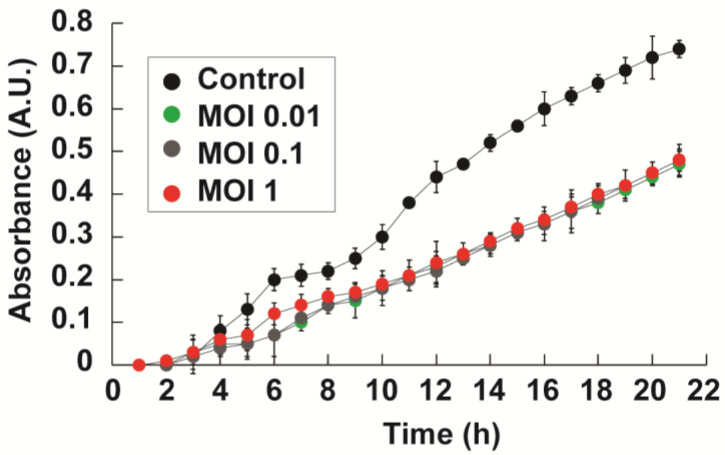
Infection curve of *P. aeruginosa* with phage M8-3 at varying MOIs. Error bars indicate standard deviations. All experiments, including controls, were performed in triplicate, with significant differences from the growth of the control observed at all time points (α = 0.05).

In preliminary co-culture experiments, we evaluated both M8-2 and M8-3 for their capacity to inhibit the growth of a multidrug-resistant *P. aeruginosa* strain. While both phages exhibited lytic activity in these initial tests, phage M8-3 consistently produced a more pronounced growth inhibition profile under the tested conditions. Therefore, we selected M8-3 as the representative phage for detailed infection curve analysis (Figure 7). To evaluate whether the cellular density in infection assays influences the lysogeny process of bacteriophages, an infection curve was generated for *P. aeruginosa* in mid-logarithmic phase (OD_600_ = 6.5 × 10^8^ CFUs/mL). The effect of phage M8-3 on bacterial growth dynamics at three multiplicities of infection (MOIs) was assessed. As shown in Figure 7, *P. aeruginosa* cultures exposed to phage M8-3 exhibited a statistically significant growth lag compared to the control (Tukey’s test, α = 0.05). This delayed growth phase suggests the effective suppression of bacterial proliferation by the phage at all tested MOIs, while still maintaining exponential growth patterns. The results highlight the impact of the phage presence on host cell dynamics, which is critical for optimizing therapeutic phage applications.

### 2.7. Viral Particle Concentration via Batch Culture Amplification

Given the relatively low viral concentrations obtained from lysed plate amplifications, batch culture amplification was employed to enhance the phage yield. This approach followed a rational amplification protocol, whereby the total viral concentration obtained inversely correlates with the initial concentration of the phage–bacteria mixture in the infection assay. To maximize viral production, an infection assay was conducted at a low MOI of 0.1. The results of this batch amplification process are presented in Table 3, revealing significantly elevated viral titers compared to lysed plate methods (Table 2). Specifically, yields of 8.0 × 10^7^ PFUs/mL for phage M8-2 and 1.03 × 10^9^ PFUs/mL for phage M8-3 were achieved, underscoring the efficiency of batch culture for scaling up phage production.

This batch culture method offers a robust strategy for amplifying phage titers, which is essential for therapeutic applications that require high phage densities to ensure effective bacterial suppression. The significant increase in viral yield demonstrates the feasibility of this approach for large-scale phage production.

### 2.8. Viral DNA Analysis and SDS-PAGE Analysis of Structural Proteins

The viral DNA extracted from phages M8-2 and M8-3 was analyzed via agarose gel electrophoresis to assess genome integrity. As shown in Figure S1, the viral DNA stocks for both phages (Lanes 2 and 4) displayed faint, slightly degraded bands, indicating the partial fragmentation of the viral DNA. Lane 1 contains the intact viral DNA from phage M8-2, and Lane 3 shows the intact viral DNA from phage M8-3. These faint bands suggest some degree of degradation during the extraction process, yet provide sufficient material for subsequent analyses.

To further characterize the structural components of these phages, an SDS-PAGE analysis was conducted to visualize the protein profiles. As illustrated in Figure S2, both phages exhibited similar banding patterns, with five distinct structural proteins observed. These proteins corresponded to approximate molecular weights of 95 kDa, 65 kDa, 50 kDa, 40–45 kDa, and 30 kDa, respectively, indicating the presence of conserved structural elements among the two phage isolates.

Although SDS-PAGE provides only a preliminary assessment of viral protein profiles, it served as an initial step to confirm the presence of structural proteins in phages M8-2 and M8-3. To complement this basic protein visualization, we performed whole-genome sequencing (WGS), which offers a deeper understanding of the gene content and potential protein functions. We acknowledge that SDS-PAGE alone has limitations compared to contemporary proteomic and genomic approaches and that future work should involve more sensitive techniques for definitive protein identification and characterization.

### 2.9. Genomic Data Quality and Sequencing Analysis

Following DNA extraction, next-generation sequencing (NGS) was performed on the viral genomes of phages M8-2 and M8-3. Quality metrics from raw NGS data were analyzed to ensure high-quality sequencing reads. Figures S3 and S4 present the Phred quality scores across reads for both genomes, with the majority of reads showing values above Q30, indicating high base-calling accuracy. Additionally, minimal fluctuations in the base composition and “N” content were observed, suggesting stable sequence integrity throughout the reads. Nevertheless, overrepresented and repeated sequences were detected (Figure S5) and subsequently removed before assembly.

**Figure 8:**
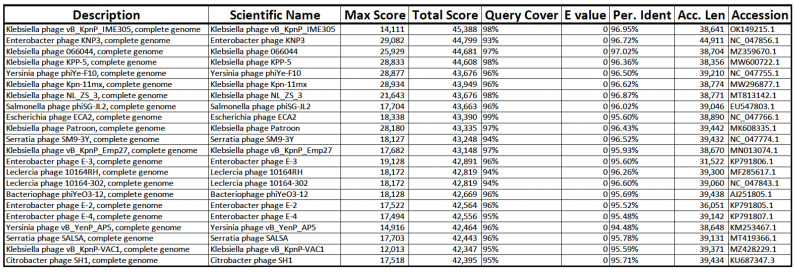
BLASTn analysis of the phage M8-2 genome. Phage *vB_KpnP_IME305* exhibits the highest similarity score, supporting its close relationship with phages infecting Gram-negative bacteria.

**Figure 9:**
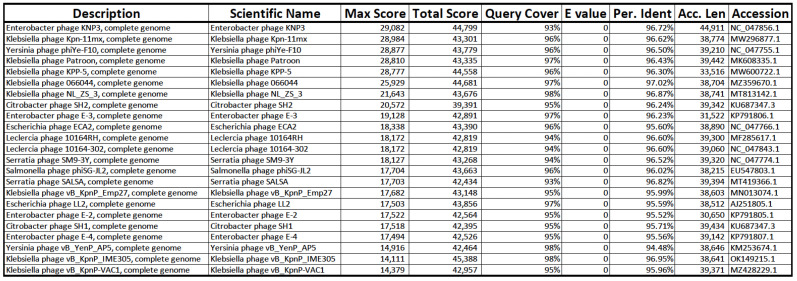
BLASTn analysis of the phage M8-3 genome. Phage KNP3 demonstrates the highest similarity, confirming the alignment results for this Gram-negative bacterial phage.

Using BLASTn VERSION+-x64-win64, the assembled genomes of phages M8-2 and M8-3 were found to share high sequence similarity (≥95%) with phages from the *Caudoviridae* family, which target Gram-negative bacterial genera, including *Klebsiella*, *Enterobacter*, *Yersinia*, *Salmonella*, *Escherichia*, and *Serratia* (Figure 8 and Figure 9). Specifically, phage M8-2 exhibited 96.95% similarity with *vB_KpnP_IME305* (Accession No.: OK149215.1) and an E-value of 0.0, while phage M8-3 showed 96.72% similarity with *KNP3* (Accession No.: NC_047856.1), also with an E-value of 0.0. These alignments provide insight into the genetic homology and potential host specificity of the isolated phages.

### 2.10. Structural Prediction of Open Reading Frames in Phage Genomes

**Figure 10:**
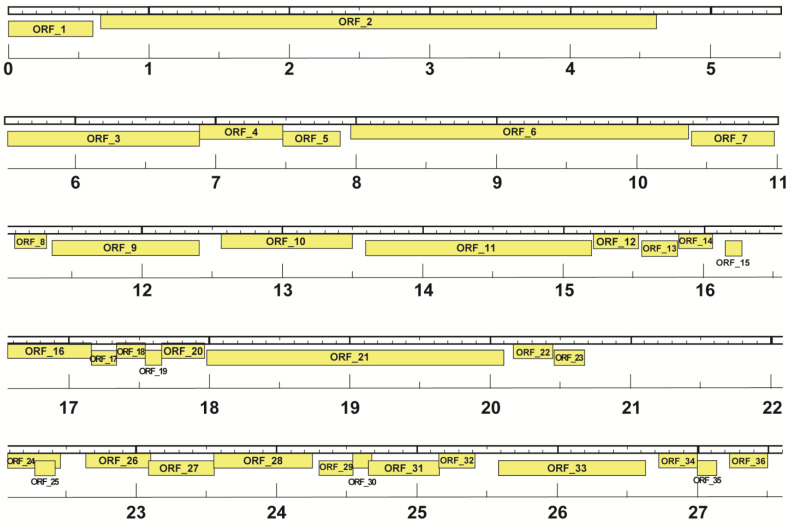
Genomic map of phage M8-2 generated by DNA Master. The Glimmer3 prediction identified 36 ORFs, all encoded on the reverse strand, exhibiting significant ORF overlap.

The M8-2 phage genome was analyzed using the Glimmer3 prediction algorithm, which identified a total of 36 open reading frames (ORFs), as detailed in Table 4. These ORFs are distributed across the genome, with all ORFs located on the reverse strand (3′→5′), indicating a high degree of genome packaging. Notably, overlapping regions were observed among specific ORFs, particularly between ORFs 15–20 and ORFs 27–32. Moreover, the algorithm revealed that ORF 25 is entirely nested within ORF 24, indicating a potential regulatory or functional interaction within these overlapping coding regions. The complete ORF map for phage M8-2 is illustrated in Figure 10, which presents a comprehensive genomic map.

**Figure 11:**
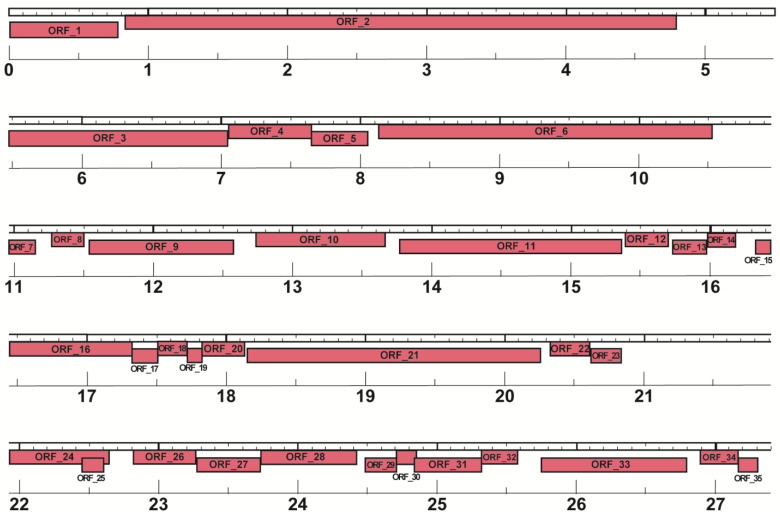
Genomic map of phage M8-3 generated by DNA Master. The Glimmer3 algorithm identified 35 ORFs, all on the reverse strand, demonstrating a high degree of overlap and packaging.

Similarly, the phage M8-3 genome was analyzed, yielding 35 ORFs, as summarized in Table 5 and visualized in Figure 11. Like phage M8-2, all ORFs in the phage M8-3 genome are located on the reverse strand (3′→5′) and show considerable overlap, reflecting a compact genome organization. This level of ORF packing suggests a highly efficient use of genomic space, which may be characteristic of bacteriophages targeting Gram-negative bacteria. The genomic map of phage M8-3 further highlights the overlap and arrangement of these ORFs, providing insights into the structural organization of this phage genome.

## 3. Discussion

Multidrug-resistant bacterial (MDRB) infections have increased dramatically in recent decades. The accelerating rate of resistance has surpassed the development of new antibiotics, necessitating alternative therapeutic approaches. Among these, bacteriophages (phages), natural predators of bacteria, have gained attention due to their high host specificity, which enables them to control bacterial populations without impacting human cells or beneficial bacteria in the microbiota, which are typically affected by antibiotics.

Phages are the most abundant entities in the biosphere [6] and are intrinsically linked to their bacterial hosts, often isolated from the same environment [20]. In this study, two phages, M8-2 and M8-3, were isolated from 10 wastewater samples collected from the Topo Chico stream. These phages demonstrated lytic potential against a multidrug-resistant *P. aeruginosa* (BMA) strain endemic to Nuevo León (Figure 2 and Figure 3). Wastewater is a productive source for isolating new phages due to its high bacterial load, which supports phage propagation [21]. Phage presence in such samples serves as an indicator of specific bacterial contamination [22]. The isolation of phages against *P. aeruginosa* suggests the prevalence of this pathogen in the collected samples, consistent with its frequent presence in wastewater [23]. Notably, no specific phage against the clinical strain of *Staphylococcus aureus* was isolated, likely due to the heterogeneous and dynamic nature of wastewater, where microbial populations fluctuate [24].

The temperate nature of phages M8-2 and M8-3 is evidenced by the turbid plaques they produced that were attributed to partially lysed cells and accumulated plasma membranes in the growth zones of lysogens. The literature suggests that temperate phages are more commonly isolated from environments with lower nutrient concentrations, as these conditions may favor lysogeny over lysis [25]. The dynamic nature of wastewater phage populations supports the isolation of diverse phages specific to multidrug-resistant bacteria, as demonstrated by other studies. For instance, Bhetwal et al. [26] reported the isolation of 67 phages from river water samples contaminated with fecal material, including the broad-spectrum phage ΦSER1, which infects multiple pathogenic strains. Similarly, Shitthisak et al. [27] developed a cocktail of five phages from wastewater that successfully targeted 42 strains of multidrug-resistant *Acinetobacter baumannii*. These findings underscore the potential for isolating broad-spectrum phages from complex environments and highlight the need to assess the lytic properties of isolated phages, like M8-2 and M8-3, against various clinical *P. aeruginosa* strains to determine their therapeutic applicability [28].

In this study, we initially used a multidrug-resistant *P. aeruginosa* strain for phage isolation to maximize the relevance to clinical MDRB infections. For subsequent characterization and amplification, the *P. aeruginosa* ATCC 27853 strain was employed. This strategy minimized the risk of horizontal gene transfer of virulence or resistance genes to the phage genome, ensuring safer therapeutic applications [29]. Amplification in a non-resistant strain also allowed the development of clonal phage populations, enhancing lytic efficiency against the ATCC strain (Figure 7) and producing phages with consistent biological properties, such as the growth rate and plaque morphology, which are critical for therapeutic reliability [30].

Further biological characterization, including one-step growth curves, is needed to confirm that amplification in this strain has not altered the phages’ biological characteristics. The balance between lysogeny and lysis in temperate phages like M8-2 and M8-3 depends on several factors, with the multiplicity of infection (MOI) being a key determinant. At higher MOIs, lysogeny is more likely, as the increased viral genome copy number within host cells promotes the accumulation of proteins that suppress lytic functions [31,32]. Optimal viral yields were achieved using lower MOIs during rational amplification, as evidenced by the linear growth of infection curves (Figure 7) [33]. This proportional growth suggests that resistant bacterial populations did not emerge, as there were no changes in bacterial growth rate in the presence of the phages [34].

The faint protein bands observed in the SDS-PAGE analysis of both phages likely result from low viral concentrations in the gel. Different studies have noted that titers above 1.0 × 10^9^ PFUs/µL are typically needed for optimal protein visualization, while the viral concentrations achieved here were lower, at 8.0 × 10^5^ PFUs/µL and 1.0 × 10^7^ PFUs/µL for phages M8-2 and M8-3, respectively. Improved protein visualization could be achieved with more sensitive stains, such as silver nitrate.

Genome sequencing of phages M8-2 and M8-3 revealed similar genetic architectures, suggesting gene conservation driven by shared ecological pressures or limited genetic divergence. Future bioinformatic analyses of orthologous genes will be essential to understand the evolutionary relationships between these phages and related isolates. The phages isolated here belong to the *Caudoviridae* order, as inferred from sequence similarity (>95%) with other members of this order (Figure 10 and Figure 11). Members of the Caudoviridae family generally possess double-stranded DNA genomes, similar to those sequenced here. For a definitive taxonomic classification, further bioinformatics work is required to confirm the functional assignments of structural proteins, such as those associated with the base plate, tail, and capsid stabilization. Additionally, protein similarity analyses with closely related phages could determine minor taxonomic classifications, as phages sharing >40% similarity in structural proteins can often be grouped within the same genus.

The diversity of bacterial pathogens in MDR infections suggests that a phage cocktail combining multiple phages with different host specificities could enhance therapeutic efficacy. Phages M8-2 and M8-3 could be tested in combination with other phages to expand their host range and explore potential synergistic effects on *P. aeruginosa* and other pathogens. The high genetic similarity between phages M8-2 and M8-3 suggests they may have co-evolved with local bacterial strains. Longitudinal studies of the co-evolution of phages and *P. aeruginosa* in wastewater could offer insights into phage adaptability and persistence, which are crucial for evaluating the long-term viability of these phages as therapeutic agents. Additional testing of a panel of clinical *P. aeruginosa* isolates could clarify the host range of phages M8-2 and M8-3. Determining whether these phages can target multiple *P. aeruginosa* strains or other Gram-negative bacteria would enhance their therapeutic utility and inform phage therapy protocols to mitigate bacterial resistance. Given the role of biofilms in chronic infections and their resistance to antibiotics, assessing the biofilm degradation capacity of phages M8-2 and M8-3 is essential. If these phages exhibit anti-biofilm activity, it would significantly increase their therapeutic value, especially for treating chronic *P. aeruginosa* infections.

Looking ahead, a comprehensive functional annotation of the open reading frames (ORFs) in phages M8-2 and M8-3 will be crucial for delineating the precise molecular mechanisms governing their lytic activity and host specificity. In parallel, expanding the host-range assessment to encompass additional clinically significant Gram-negative pathogens (e.g., *Klebsiella pneumoniae* and *Acinetobacter baumannii*) would help clarify the breadth of their therapeutic utility. Such efforts would further inform the development of multi-phage cocktails aimed at tackling diverse multidrug-resistant infections and provide greater confidence in the potential clinical application of these locally sourced bacteriophages.

The application of temperate phages, such as M8-2 and M8-3, in phage therapy (PT) remains a topic of debate, primarily because lysogeny can sometimes enhance bacterial fitness rather than inhibit it [35]. However, temperate phages can also integrate into the bacterial genome in ways that disrupt specific virulence genes [36,37] or mobility-associated genes [38,39], potentially reducing the pathogenicity of the host. This characteristic may represent an indirect therapeutic benefit, as the specific integration of a prophage could lead to an attenuation of bacterial virulence.

Temperate phages that follow pseudolysogeny, wherein the viral genome exists as an episomal element without integrating into the host genome, offer additional promise for PT. It has been demonstrated that the jumbo lysogenic phage “PA5oct”, isolated from wastewater, exhibits anti-biofilm activity and significantly reduces the levels of virulence factors like pyocyanin, pyoverdine, and motility in *P. aeruginosa*. In resistant strains, the phage remained inside the cell as a pseudolysogen, lowering pathogenicity in vivo and enhancing immune clearance by inducing an inflammatory response that facilitates monocyte-mediated elimination. This example suggests that temperate phages like M8-2 and M8-3 might be harnessed for similar benefits, particularly in complex infections where direct lysis may not be feasible.

Advances in genetic engineering have made it possible to modify temperate phages to enhance their therapeutic potential by removing their lysogenic modules. Dedrick et al. [40] utilized the Bacteriophage Recombineering of Electroporated DNA (BRED) technique to delete repressor genes in the temperate phages ZoeJ and BPs, both targeting *Mycobacterium smegmatis* and *Mycobacterium abscessus*. By disabling the lysogenic pathway, these modified phages exhibited enhanced lytic capabilities, proving effective in a therapeutic cocktail that led to a clinical improvement and infection resolution in a patient with a disseminated *M. abscessus* infection. A similar approach could be explored with phages M8-2 and M8-3, potentially transforming them into obligate lytic phages to avoid the risks associated with lysogeny.

In addition to their standalone therapeutic potential, phages like M8-2 and M8-3 could also be combined with antibiotics to leverage synergistic effects. Some studies have shown that certain antibiotics can increase the susceptibility of bacterial cells to phage infection by altering cell wall permeability or by triggering stress responses that facilitate phage entry. This synergy may help overcome partial resistance, reduce the effective antibiotic dose required, and slow the emergence of further resistance. Notably, the delayed regrowth observed in Figure 7 suggests that monotherapy with phage M8-3 may be insufficient for complete bacterial eradication, further underscoring the potential need for combination therapies or phage cocktails. Future studies could investigate whether combining phages M8-2 and M8-3 with selected antibiotics enhances their lytic efficiency against *P. aeruginosa*.

The use of phages sourced from environments like wastewater necessitates thorough characterization and safety evaluations. While phages are typically host-specific and do not infect human cells, temperate phages carry a risk of horizontal gene transfer, which could inadvertently spread antibiotic resistance or virulence factors. Ensuring that phages like M8-2 and M8-3 lack undesirable genes through whole-genome sequencing and bioinformatic analyses is essential before their clinical application. Moreover, evaluating their stability and activity under physiological conditions would help assess their viability as therapeutic agents.

Although our next-generation sequencing and bioinformatic analyses did not reveal known antibiotic resistance or virulence genes in phages M8-2 and M8-3, we acknowledge that NGS alone does not constitute a complete safety evaluation. Future studies should incorporate advanced screening against comprehensive databases, electron microscopy for morphological characterization, expanded host range assays to confirm specificity, and in vivo testing to ensure the absence of adverse reactions or horizontal gene transfer events. These additional evaluations will be crucial for verifying the safety profiles of these phages prior to therapeutic use.

Furthermore, given the potential temperate behavior of phages M8-2 and M8-3, it is important to evaluate whether their genomic integration could disrupt or suppress *P. aeruginosa* virulence factors. Such integration events might reduce bacterial pathogenicity and thereby enhance therapeutic outcomes, but they also raise the possibility of inadvertent horizontal gene transfer if any resistance determinants reside near integration sites. Future studies aimed at precisely mapping phage integration loci and validating the absence of transferred resistance or toxicity-associated genes will be critical to ensure the safe and effective use of these phages.

This study isolated and characterized two temperate phages, M8-2 and M8-3, with lytic potential against multidrug-resistant *P. aeruginosa*. These phages offer promising prospects for phage therapy, particularly in light of their specificity and ability to coexist within complex bacterial communities. While temperate phages like M8-2 and M8-3 require careful consideration due to their potential for lysogeny, they could be harnessed through genetic engineering or combined with other therapies to mitigate these risks. Additionally, their potential roles in biofilm degradation, host range expansion through phage cocktails, and application in combination therapies merit further exploration. With the appropriate safety and efficacy assessments, phages M8-2 and M8-3 could contribute as valuable tools in combating MDRB infections, particularly those caused by *P. aeruginosa*, an increasingly prevalent pathogen in healthcare settings.

An equally critical aspect for validating the therapeutic potential of phages M8-2 and M8-3 is assessing their efficacy against a broader range of extensively drug-resistant (XDR) or pan-drug-resistant (PDR) *P. aeruginosa* strains. Demonstrating robust lytic activity under these most challenging clinical conditions would provide stronger evidence of their suitability for real-world therapeutic applications. Although we have primarily tested a single multidrug-resistant clinical isolate in this study, future work should encompass a more diverse panel of highly resistant strains to fully ascertain the clinical scope of these locally sourced phages.

Notably, our work introduces a novel perspective on how locally sourced temperate phages, such as M8-2 and M8-3, can be leveraged to address multidrug-resistant pathogens in a specific geographic region. By demonstrating the isolation and genomic profiling of phages from wastewater samples in the Monterrey Metropolitan Area (MMA), our study highlights the potential for discovering customized phage solutions that align with regional microbial ecologies. This local approach may ultimately lead to more effective and sustainable phage therapies, reducing both the time and costs associated with developing broadly targeted antimicrobial strategies. In doing so, we provide a promising model for harnessing environmental phage diversity to combat escalating antibiotic resistance challenges.

In addition to our current findings, future studies should include one-step growth curve analyses and burst size determinations to elucidate the phage replication dynamics. These experiments will provide insights into the phages’ infection efficiency and speed, which are crucial for both laboratory optimization and clinical application. Moreover, extended time course or passaging experiments could clarify whether bacterial populations adapt or develop resistance to phages M8-3 and M8-2 over the long term, an essential consideration for predicting phage durability in therapeutic settings. Equally important is evaluating their stability and lytic activity under various pH and temperature conditions, as these parameters dictate whether the phages can retain efficacy across diverse environments. These characterizations represent essential future avenues of inquiry for confirming the robustness and therapeutic viability of these phages.

## 4. Materials and Methods

### 4.1. Bacterial Strains

A clinical isolate of multidrug-resistant *P. aeruginosa* was generously provided by the Bacteriology Department of Hospital de San Vicente, Monterrey, Nuevo León, Mexico. Additionally, a reference strain, *P. aeruginosa* ATCC 27853, was utilized for comparison. Working stocks were prepared by inoculating 200 µL of the preserved stock of each strain into 5 mL of LB broth (Difco, Detroit, MI, USA), followed by an incubation at 37 °C with agitation at 150 rpm for 16 h. After incubation, cultures were stored at 4 °C until required.

### 4.2. Sample Collection and Bacteriophage Isolation

Environmental samples were collected to isolate bacteriophages from natural sources. Ten water samples, each in a sterile 50 mL tube (Wuxi NEST Biotechnology Co., Ltd., Wuxi, China), were taken from the Topo Chico stream at the intersection of Av. Universidad and Av. Jorge Treviño, San Nicolás de los Garza, Nuevo León, Mexico. Sampling was conducted at 8:00 am under conditions of a 13 °C ambient temperature and 78% relative humidity. Collection tubes were rinsed three times with stream water before sample collection to reduce external contamination. Samples were transported to the laboratory under refrigeration and subsequently processed.

For phage enrichment, each sample was added to 5 mL of LB broth (Difco, Detroit, MI, USA) and incubated for 2 h at 150 rpm at 37 °C. Samples were then chilled for 1 h at 4 °C, and the total volume was filtered through 0.22 µm filters (Whatman, Maidstone, Kent, UK) to remove bacterial cells, leaving only potential phage particles. Filtered samples were stored in sterile 15 mL tubes (Wuxi NEST Biotechnology Co., Ltd., Wuxi, China) at 4 °C for subsequent analyses.

### 4.3. Preparation of the Bacterial Inoculum and Double-Layer Agar (DLA) Technique

To prepare the bacterial inoculum, the pre-enriched bacterial culture was diluted 1:20 in LB broth and incubated at 37 °C with agitation at 150 rpm until reaching an optical density (OD_600_) of 0.6 ± 0.02. The DLA method was adapted from Adams et al. [41], with modifications. Briefly, 125 µL of the *P. aeruginosa* strain was mixed with 500 µL of filtered sample in 10 separate 15 mL tubes (one for each sample). After a 20 min incubation, 5 mL of LB soft agar (0.5% agar supplemented with 5 mM CaCl_2_) was added to each mixture, inverted gently three times, and poured onto Petri dishes containing an LB base agar layer (2% agar). Plates were incubated at 37 °C for 24 h. Lytic zones observed on the agar (indicating phage activity) were collected by puncturing the agar with sterile tips and resuspending them in SM buffer (100 mM NaCl, 8 mM MgSO_4_·7H_2_O, 50 mM Tris-HCl, pH 7.5). Samples were stored at 4 °C.

### 4.4. Viral Confirmation by a Spot Test

For further confirmation, the DLA technique was employed as a spot test. Starting with the working inoculum, 125 µL of the multidrug-resistant *P. aeruginosa* strain was added to 5 mL of LB soft agar (0.5% agar with 5 mM CaCl_2_) and poured onto an LB base agar plate. After the agar dried, 10 µL from each of the eleven collected lytic plates was spotted on the DLA. As a negative control, 10 µL of SM buffer was also spotted. Plates were incubated at 37 °C for 16 h, after which clear lytic zones were noted, confirming the presence of phage activity. The phages were subsequently resuspended in SM buffer and stored at 4 °C.

### 4.5. Quantification of Viral Titers

To quantify phage titers, 125 µL of the bacterial working inoculum was distributed into 24 separate 15 mL tubes. Serial dilutions (10^−1^ to 10^−8^) of phages M7-1, M8-2, and M8-3 were prepared in SM buffer, with 10 µL from each dilution added to each tube containing the bacterial strain. After a 20 min incubation, 5 mL of LB soft agar (0.5% agar with 5 mM CaCl_2_) was added, inverted three times, and poured onto Petri dishes with LB base agar. Plates were incubated at 37 °C for 16 h, and plaque-forming units (PFUs) were counted on plates with 20–200 PFUs to calculate the viral titer using the following formula:

$$UFP\cdot {mL}^{-1}=\frac{PFU\;on\;plate\;}{Dilution\;volume\;in\;µL\;aggregate}\times \frac{1000\;µL}{1\;mL}\times Dilution\;factor$$

Single lytic plaques were selected from the final dilution that showed PFUs, resuspended in SM buffer, and stored at 4 °C. The dilution, titration, and collection process were repeated three times to ensure a clonal phage population.

### 4.6. Bacteriophage Amplification by the Lysed Plate Method

To amplify the bacteriophage stocks of M8-2 and M8-3, a double-layer agar (DLA) assay was performed, adapting the methodology outlined by Bonilla et al. [42], with modifications. Briefly, 125 µL of a *P. aeruginosa* bacterial strain was inoculated into a sterile 15 mL tube, followed by the addition of 10 µL of either the M8-2 or M8-3 phage stock. The phage–bacteria mixture was then incubated at 37 °C for 20 min to promote the efficient adsorption of the phages onto the bacterial cells.

After the adsorption phase, 3 mL of LB soft agar (0.5% agar) supplemented with 5 mM CaCl_2_ and 5 mM MgCl_2_ was added to the tube. The contents were gently inverted three times to ensure thorough mixing and then poured evenly onto Petri dishes pre-coated with LB base agar (2% agar). Once the soft agar layer had solidified, the plates were incubated at 37 °C for 24 h to facilitate plaque formation and phage amplification.

Following the incubation, the soft agar layer containing lytic plaques was carefully collected from each Petri dish and transferred into 50 mL sterile tubes containing 3 mL of SM buffer supplemented with 5 mM CaCl_2_ and 5 mM MgCl_2_. The tubes were briefly vortexed for 10 s to ensure an even suspension of viral particles and then centrifuged at 4000× *g* for 20 min at 4 °C to remove cellular debris. The supernatant containing the amplified phages was carefully collected and passed through a 0.22 µm filter (Whatman) to further purify the phage preparation and remove any residual bacterial cells.

Subsequently, the viral titer was determined by performing a spot test viral titer assay on the DLA of both the M8-2 and M8-3 phage samples. Serial dilutions (from 10^−1^ to 10^−6^) of each phage sample were prepared in SM buffer, adjusting each dilution to a final volume of 100 µL. From each dilution, 10 µL was spotted onto double-layer agar plates. After allowing the spots to dry, the plates were incubated at 37 °C for 20 h. The resulting plaques were counted, and the viral titer was quantified based on the dilution factors and plaque-forming units (PFUs) per mL, following standard DLA quantification methods.

### 4.7. Optimization of the Viral Yield and Rational Amplification

To determine the optimal dilution factor yielding the highest viral concentration, soft agar from each Petri dish—corresponding to the specific viral titer of each sample and dilution factor—was collected and transferred into sterile 50 mL tubes. Each tube contained 3 mL of SM buffer supplemented with 5 mM CaCl_2_ and 5 mM MgCl_2_ to facilitate phage suspension.

The tubes were vortexed for 10 s to ensure thorough mixing of the phage particles within the buffer. Following vortexing, the tubes were centrifuged at 4000× *g* for 20 min at 4 °C to separate viral particles from residual agar and bacterial debris. The supernatant from each tube, representing a different dilution factor, was carefully collected and filtered through a 0.22 µm filter (Whatman) to obtain a purified phage solution for subsequent quantification.

Using the double-layer agar (DLA) technique, the viral concentration was quantified in triplicate for each dilution factor to ensure accuracy and reproducibility. For each dilution, plaque-forming units (PFUs) were counted on the resulting plates, and the viral yield was determined by correlating the PFU count with the initial dilution factor. This approach enabled the identification of the dilution factor that produced the highest viral yield, optimizing the conditions for the subsequent amplification and application of the phages.

Building on these findings, we then performed the rational amplification of phages M8-2 and M8-3 using the optimized dilution factor. Briefly, 1250 µL of the *P. aeruginosa* working culture was divided into two sterile 50 mL tubes, each containing 100 µL of the phage suspensions diluted at a 10^−1^ factor. This mixture was incubated at 37 °C for 20 min to promote optimal phage adsorption to the bacterial cells. Following this adsorption period, 30 mL of LB soft agar (0.5% agar) supplemented with 5 mM CaCl_2_ and 5 mM MgCl_2_ was added to each tube, and the suspension was gently inverted three times to ensure even mixing.

Subsequently, 3 mL aliquots of the phage–bacteria mixture were dispensed onto Petri dishes pre-coated with LB base agar (2% agar). The plates were incubated for 20 h at 37 °C to allow for visible plaque formation indicative of phage lytic activity. After the incubation, the soft agar from each plate was collected into a single 50 mL tube, representing the amplified sample. The viral titers were then determined using the double-layer agar (DLA) technique.

### 4.8. Phage Culture with Bacteria

For further studies, a pre-inoculum of *P. aeruginosa* was prepared by incubating 5 mL of LB broth containing the strain at 37 °C with agitation at 150 rpm for 20 h. The resulting culture was then diluted 1:10 in LB broth supplemented with 5 mM CaCl_2_ and 5 mM MgCl_2_ and incubated until an optical density (OD_600_) of 0.6 ± 0.2 was achieved. Using this inoculum, infection assays were set up in 96-well microplates at multiplicities of infection (MOIs) of 10, 1, and 0.1 for phage M8-3. Each condition was performed in triplicate. The microplates were incubated statically at 37 °C, with brief shaking for 10 s prior to the OD_600_ measurement every hour for 21 h using a MultiskanGo microplate reader (Thermo Fisher, Waltham, MA, USA) to monitor the growth dynamics in the presence of the phage at varying MOIs.

### 4.9. Viral Particle Concentration

For the production of concentrated phage particles, infection assays were prepared with both M8-2 and M8-3 phages at an MOI of 0.1, based on the *P. aeruginosa* inoculum. The phage–bacteria mixtures were allowed to incubate at 37 °C for 15 min to promote phage adsorption, and then inoculated into flasks containing 750 mL of LB broth with 5 mM CaCl_2_ and 5 mM MgCl_2_. The cultures were agitated at 150 rpm at 37 °C for 20 h, at which point 1 mL of chloroform per 100 mL of culture was added to each flask. The flasks were shaken for an additional 10 min at 150 rpm to lyse any remaining bacterial cells and release phage particles. The culture suspensions were subsequently centrifuged at 4000× *g* for 20 min at 25 °C, and the resulting supernatants were collected and stored in sterile 1 L flasks at 4 °C.

To further concentrate the viral particles, the method of Armon, Arella and Payment (1988) [43] was modified as follows: Tween 80 was added to a final concentration of 3% (*v*/*v*) and (NH_4_)_2_SO_4_ to 35% (*m*/*v*). The mixture was stirred at 2000 rpm until (NH_4_)_2_SO_4_ was fully dissolved. The suspension was centrifuged at 3000× *g* for 20 min at 4 °C, after which the upper layer (containing concentrated viral particles) was carefully collected and transferred to 250 mL Pyrex vials (Corning Inc., Corning, NY, USA). The collected films were then resuspended in 50 mL of SM buffer and quantified.

### 4.10. SDS-PAGE and Viral DNA Extraction

To prepare for SDS-PAGE and viral DNA extraction, each viral stock was filtered through a 0.22 µm Whatman filter and sterilized as per the method described by Kerketta et al. (2014) [44], with modifications. Ten milliliters of the filtered viral stock were combined with four volumes of −20 °C acetone in sterile 50 mL tubes. These tubes were wrapped in aluminum foil and incubated at −20 °C for one hour. The samples were then centrifuged at 5000× *g* for 20 min at 4 °C, a step that was repeated twice to ensure purity. After centrifugation, the supernatant was decanted, and the viral pellet was resuspended in 1 mL of SM buffer.

The resuspended phages underwent dialysis in two stages using a 34 mm membrane with a 14 kDa cut-off (Biobasic Inc., Markham, ON, Canada). Dialysis was performed at a 1:1000 ratio in SM buffer (chilled to 4 °C) under constant magnetic stirring at 200 rpm. The first dialysis stage lasted for 24 h, followed by buffer replacement and an additional two-hour dialysis. Dialyzed samples were concentrated to a 1:1000 volume and stored at 4 °C in 1.5 mL tubes until needed.

For viral DNA extraction, 1 mL of the dialyzed phage suspension was processed using the Phage DNA Isolation Kit (Norgen Biotek, Thorold, ON, Canada) following the manufacturer’s instructions. The DNA concentration was measured with a BioSpec-nano spectrophotometer (Shimadzu Co., Kyoto, Japan), and DNA integrity was verified by electrophoresis on a 0.8% agarose gel.

To visualize the structural proteins of the dialyzed phages, SDS-PAGE was conducted, following the protocol by Beer and Speicher (2018) [45], with modifications for optimization. Each phage sample (28 µL) was transferred into a 0.2 mL tube and combined with 4 µL of 4× SDS loading buffer, comprising 1.5 M Tris (pH 6.8), 20% SDS, 40% glycerol, 0.4 M DTT, and 1% bromophenol blue (*m*/*v*) in deionized water. This mixture was heated at 100 °C for 10 min to ensure the complete denaturation of viral proteins.

The prepared samples were then loaded onto an SDS-PAGE gel system consisting of a 4% concentrating (stacking) gel and a 12% separating (resolving) gel. Electrophoresis was carried out at 180 V for 60 min to achieve the optimal separation of protein bands. Following electrophoresis, the gel was washed three times with double-distilled water to remove the residual SDS and buffer components.

To visualize the proteins, the gel was immersed in a staining solution containing 50% absolute methanol (*v*/*v*), 0.1% Coomassie Brilliant Blue R-250 (*w*/*v*), and 10% absolute acetic acid (*v*/*v*). The gel was gently agitated overnight at 60 rpm using an Orbit 1000 shaker (Labnet International, Inc., Edison, NJ, USA) to allow thorough and uniform staining of the protein bands.

After staining, the gel was washed three times with double-distilled water to remove the excess stain, followed by immersion in a de-staining solution comprising 15% absolute methanol (*v*/*v*) and 10% absolute acetic acid (*v*/*v*). The gel was further agitated in the de-staining solution for an additional two hours at 60 rpm in the Orbit 1000 shaker to enhance the band clarity. This step ensured the optimal visualization of structural proteins, aiding in the assessment of the protein composition within the phage samples.

### 4.11. Next-Generation Sequencing (NGS) of Viral Genomes 

Prior to sequencing, viral DNA from both phage samples was concentrated to ensure an optimal quality and quantity for high-throughput sequencing. To achieve this, each DNA sample was treated with a 1/10 volume of 3 M sodium acetate (pH 5.0) and 3 volumes of absolute ethanol (99%). The mixture was thoroughly homogenized by vortexing and subsequently incubated at −20 °C for 24 h to enhance DNA precipitation.

Following the incubation period, the samples were centrifuged at 16,000 rpm for 30 min to pellet the DNA. After centrifugation, the supernatant was carefully decanted, and the DNA pellet was air-dried for 20 min to remove any residual ethanol. The dried DNA pellet was then resuspended in 20 µL of 1× TE buffer (pH 8.0), which contained 10 mM Tris-HCl and 1 mM EDTA-Na_2_, to protect against degradation and maintain stability.

Next-generation sequencing (NGS) was performed by Macrogen (Seoul, Republic of Korea) using the TruSeq DNA kit (Illumina Inc. San Diego, CA, USA). A throughput of 1 GB of raw data per genome was requested, providing sufficient coverage for a comprehensive genome analysis. All bioinformatic analyses, including quality control, assembly, and annotation, were conducted on the Galaxy CPT Public platform (https://cpt.tamu.edu/galaxy-pub, accessed on 20 October 2022) utilizing a standardized workflow for viral genome assembly and data analysis.

### 4.12. Analysis and Quality Control of Genetic Data

To assess the quality and accuracy of the raw sequencing data and to assemble the genomes of phages M8-2 and M8-3, a comprehensive workflow was executed using the “Phage Genome Assembler v2021.01” suite available on the Galaxy CPT Public platform. This workflow included a series of bioinformatics tools designed to ensure a high-quality genome assembly.

Initially, Phred quality scores were evaluated across both sequencing files to assess the read reliability. Sequencing adapters and low-quality reads (Phred scores < Q33) were removed to improve the fidelity of the assembly process. For this pre-processing, the Trimmomatic tool (Bolger, Lohse and Usadel, 2014) [46] was employed, ensuring that only high-quality reads (Phred score > 33) were retained for subsequent assembly steps.

The remaining high-quality reads were then assembled using SPAdes (Bankevich et al., 2012) [47], with parameters specifically configured to handle only reads meeting the defined quality threshold. Upon completion of the assembly, the resulting contig and scaffold files were extracted. The consistency of the assembly was verified by comparing the lengths of NODES across both genome files, ensuring the alignment and structural similarity between the two assembled genomes.

The longest contig, designated as NODE_1, was identified in both phage genomes, with lengths of 27,610 bp for phage M8-2 and 27,404 bp for phage M8-3. Each NODE_1 contig was subjected to a megaBLASTn analysis (https://blast.ncbi.nlm.nih.gov/Blast.cgi, accessed on 15 December 2022) to assess similarity with known viral genomes. A stringent E-value threshold (≤10^−10^) was applied to identify significant matches, providing insights into the genetic relationships of the isolated phages with previously characterized viruses.

Further annotation was performed using DNA Master (version 5.0.2), which facilitated the application of Glimmer3, a gene prediction tool, to identify open reading frames (ORFs) within the viral genomes. For accuracy, the standard genetic code for Bacteria and Archaea was used, with TTG and GTG included as possible alternative start codons. After the ORF prediction, a detailed genomic map of each phage was generated, depicting the identified ORFs and their relative positions within the genome.

## 5. Conclusions

The isolation and characterization of bacteriophages M8-2 and M8-3 mark a significant step forward in the quest for alternative treatments against multidrug-resistant *P. aeruginosa*, a critical-priority pathogen that poses a severe threat to global health due to its high prevalence in healthcare environments and resistance to last-resort antibiotics. Our study demonstrates that phages isolated from local environmental sources, such as the Topo Chico stream, possess lytic potential against both antibiotic-resistant and antibiotic-sensitive strains of *P. aeruginosa*, regardless of whether the host used during isolation was resistant or sensitive to antibiotics. This finding is promising, as it suggests that phages isolated in this manner can be versatile in their application, potentially providing a therapeutic option that could be effective against heterogeneous bacterial populations with varying antibiotic resistance profiles that are commonly found in clinical settings.

Despite these promising findings, additional research is required to validate their therapeutic efficacy and safety. A functional annotation of open reading frames (ORFs), assessment of their broader host range, and detailed genomic analyses are needed to rule out undesirable genes and to understand their potential for disrupting bacterial virulence. Establishing optimal dosing parameters, evaluating synergistic effects with antibiotics, and confirming their stability in vivo will be crucial for eventual clinical applications. Collectively, our results highlight phages M8-2 and M8-3 as viable candidates for localized, sustainable phage therapy, underscoring the broader potential of bacteriophages to combat the rising threat of antibiotic-resistant pathogens.

## Figures and Tables

**Table 1:** Determination of the viral titers in phage stocks.

Phage	PFUs	Inoculated Volume	Dilution Factor	Viral Titer
M8-2	61	10 µL	10 ^4^	6.1 × 10^7^ PFUs/mL
M8-3	26	10 µL	10 ^4^	2.6 × 10^7^ PFUs/mL

**Table 2:** Determination of viral titers in the samples amplified by plate lysate in the PA strain.

Phage	PFUs	Inoculated Volume	Dilution Factor	Viral Titer
M8-2	42	10 µL	10^4^	4.2 × 10^7^ PFUs/mL
M8-3	13	10 µL	10^4^	3.0 × 10^7^ PFUs/mL

**Table 3:** Viral titers obtained from the batch culture amplification of phages M8-2 and M8-3 in *P. aeruginosa* cultures.

Phage	PFUs	Inoculated Volume	Dilution Factor	Viral Titer
M8-2	128	10 µL	10^5^	8.0 × 10^7^ PFUs/mL
M8-3	80	10 µL	10^4^	1.03 × 10^9^ PFUs/mL

**Table 4:** Open reading frames (ORFs) predicted by Glimmer3 in the M8-2 phage genome.

ORF	Direction	Start	End	ORF Length (pb)	Protein Length (aa)	Protein MW (kDa)
M8-2-ORF1	R	3	611	609	203	22.32
M8-2-ORF2	R	661	4623	3963	1321	143.94
M8-2-ORF3	R	4642	6885	2244	748	85.4
M8-2-ORF4	R	6888	7481	594	198	21.35
M8-2-ORF5	R	7484	7894	411	137	15.88
M8-2-ORF6	R	7967	10,372	2406	802	90.19
M8-2-ORF7	R	10,388	10,978	591	197	22.29
M8-2-ORF8	R	11,091	11,324	234	78	7.45
M8-2-ORF9	R	11,363	12,406	1044	348	36.94
M8-2-ORF10	R	12,563	13,495	933	311	33.75
M8-2-ORF11	R	13,597	15,204	1608	536	58.76
M8-2-ORF12	R	15,215	15,535	321	107	10.93
M8-2-ORF13	R	15,562	15,813	252	84	8.88
M8-2-ORF14	R	15,818	16,063	246	82	9.37
M8-2-ORF15	R	16,156	16,269	114	38	4.16
M8-2-ORF16	R	16,248	17,162	915	305	35.03
M8-2-ORF17	R	17,159	17,341	183	61	6.83
M8-2-ORF18	R	17,338	17,547	210	70	7.28
M8-2-ORF19	R	17,547	17,663	117	39	4.43
M8-2-ORF20	R	17,663	17,965	303	101	11.1
M8-2-ORF21	R	17,982	20,096	2115	705	80.18
M8-2-ORF22	R	20,164	20,448	285	95	10.77
M8-2-ORF23	R	20,459	20,671	213	71	7.72
M8-2-ORF24	R	20,768	22,468	1701	567	63.08
M8-2-ORF25	R	22,279	22,434	156	52	5.61
M8-2-ORF26	R	22,647	23,102	456	152	17.02
M8-2-ORF27	R	23,095	23,556	462	154	17.66
M8-2-ORF28	R	23,556	24,254	699	233	26.01
M8-2-ORF29	R	24,306	24,542	237	79	8.88
M8-2-ORF30	R	24,539	24,676	138	46	5.32
M8-2-ORF31	R	24,663	25,154	492	164	18.37
M8-2-ORF32	R	25,154	25,411	258	86	9.91
M8-2-ORF33	R	25,581	26,627	1047	349	39.8
M8-2-ORF34	R	26,722	26,997	276	92	10.62
M8-2-ORF35	R	26,997	27,137	141	47	5.94
M8-2-ORF36	R	27,229	27,501	273	91	10.4

R: reverse strand (3′–5′); MW: molecular weight; pb: pair bases; aa: amino acids.

**Table 5:** Open reading frames (ORFs) predicted by Glimmer3 in the M8-3 phage genome.

ORF	Direction	Start	End	ORF Length (pb)	Protein Length (aa)	Protein MW (kDa)
M8-3-ORF_1	R	3	782	780	260	28.39
M8-3-ORF_2	R	832	4794	3963	1321	143.94
M8-3-ORF_3	R	4813	7056	2244	748	85.4
M8-3-ORF_4	R	7059	7652	594	198	21.35
M8-3-ORF_5	R	7655	8065	411	137	15.88
M8-3-ORF_6	R	8138	10,543	2406	802	90.19
M8-3-ORF_7	R	10,559	11,149	591	197	22.29
M8-3-ORF_8	R	11,262	11,495	234	78	7.45
M8-3-ORF_9	R	11,534	12,577	1044	348	36.94
M8-3-ORF_10	R	12,734	13,666	933	311	33.75
M8-3-ORF_11	R	13,768	15,375	1608	536	58.76
M8-3-ORF_12	R	15,386	15,706	321	107	10.93
M8-3-ORF_13	R	15,733	15,984	252	84	8.88
M8-3-ORF_14	R	15,989	16,183	195	65	7.46
M8-3-ORF_15	R	16,327	16,440	114	38	4.16
M8-3-ORF_16	R	16,419	17,333	915	305	35.03
M8-3-ORF_17	R	17,330	17,512	183	61	6.83
M8-3-ORF_18	R	17,509	17,718	210	70	7.28
M8-3-ORF_19	R	17,718	17,834	117	39	4.43
M8-3-ORF_20	R	17,834	18,136	303	101	11.1
M8-3-ORF_21	R	18,153	20,267	2115	705	80.18
M8-3-ORF_22	R	20,335	20,619	285	95	10.77
M8-3-ORF_23	R	20,630	20,842	213	71	7.72
M8-3-ORF_24	R	20,939	22,639	1701	567	63.08
M8-3-ORF_25	R	22,450	22,605	156	52	5.61
M8-3-ORF_26	R	22,818	23,273	456	152	17.02
M8-3-ORF_27	R	23,266	23,727	462	154	17.66
M8-3-ORF_28	R	23,727	24,425	699	233	26.01
M8-3-ORF_29	R	24,477	24,713	237	79	8.88
M8-3-ORF_30	R	24,710	24,847	138	46	5.32
M8-3-ORF_31	R	24,834	25,325	492	164	18.37
M8-3-ORF_32	R	25,325	25,582	258	86	9.91
M8-3-ORF_33	R	25,752	26,798	1047	349	39.8
M8-3-ORF_34	R	26,893	27,168	276	92	10.62
M8-3-ORF_35	R	27,168	27,308	141	47	5.94

R: reverse strand (3′–5′); MW: molecular weight; pb: pair bases; aa: amino acids.

## Data Availability

The original contributions presented in this study are included in the article. Further inquiries can be directed to the corresponding author.

## Supplementary Materials

The following supporting information can be downloaded at: https://www.mdpi.com/article/10.3390/antibiotics14020110/s1, Figure S1: Viral DNA extraction from bacteriophages M8-2 and M8-3. Electrophoresis was performed using a 0.8% agarose gel in 1X TBE buffer. M: Molecular weight marker 100–5000 bp DNA Marker Plus (Bio line); Lane 1: Viral DNA from phage M8-2; Lane 3: Viral DNA from phage M8-3; Lanes 2 and 4 represent the cell lysate stock for M8-2 and M8-3, respectively; Figure S2: SDS-PAGE analysis of structural proteins in phages M8-2 and M8-3. Polyacrylamide gel electrophoresis was conducted using a concentrating gel (4%) and a separating gel (12%). M: Broad Multi Color Pre-Stained Protein Standard (GenScript); M8-2: Structural proteins of bacteriophage M8-2; M8-3: Structural proteins of bacteriophage M8-3. The banding patterns reveal five resolved proteins with approximate molecular weights of 95 kDa, 65 kDa, 50 kDa, 40–45 kDa, and 30 kDa; Figure S3: Quality analysis with FastQC of the raw sequencing reads of phage M8-2. (A) Average Phred scores (Q-values) across the sequence length (151 bp): The line within the green area represents the quality scores (*y*-axis) across the sequencing read length (*x*-axis). (B) Distribution of Q-values across all sequencing reads. (C) Nitrogenous base content across the genome: Four parallel lines indicate no discrepancies in base calling throughout the sequencing read. (D) Content of “N” assignments (any nucleotide) across the sequencing read length; Figure S4: Quality analysis with FastQC of the raw sequencing reads of phage M8-3. (A) Average Phred scores (Q-values) across the sequence length (151 bp): The line within the green area represents the quality scores (*y*-axis) along the sequencing read length (*x*-axis). (B) Distribution of Q-values across all sequencing reads. (C) Nitrogenous base content across the genome: Four parallel lines indicate no discrepancies in base calling throughout the sequencing read. (D) Content of “N” assignments (any nucleotide) across the sequencing read length; Figure S5: Low-quality metrics by FastQC in the raw sequencing reads of phages M8-2 and M8-3. (A) Percentage of duplicated reads in the raw sequencing data of phage M8-2. (B) Overrepresented sequences in the raw sequencing data of phage M8-2: Three highly repetitive sequences were found throughout the sequencing data for phage M8-2. (C) Percentage of duplicated reads in the raw sequencing data of phage M8-3. (D) Overrepresented sequences in the raw sequencing data of phage M8-3: Three highly repetitive sequences were found throughout the sequencing data for phage M8-3.
