# Supplementary material for: Genomic Insights into and Lytic Potential of Native Bacteriophages M8-2 and M8-3 Against Clinically Relevant Multidrug-Resistant Pseudomonas aeruginosa
Source: Antibiotics (Basel). 2025 Jan 21;14(2):110. doi: 10.3390/antibiotics14020110 (PMC11851605; doi:10.3390/antibiotics14020110)
Supplement: Supplementary file 1 [file antibiotics-14-00110-s001.zip › antibiotics-3346356-supplementary.pdf]

Genomic Insights into and Lytic Potential of Native Bacteriophages M8-2 and M8-3 Against Clinically Relevant Multidrug-Resistant *Pseudomonas aeruginosa*

Francisco Ricardo Rodríguez-Recio [1,2], Javier Alberto Garza-Cervantes [1,2], Francisco de Jesús Balderas-Cisneros[1,2] and José Rubén Morones-Ramírez [1,2,]*

[1] Facultad de Ciencias Químicas, Universidad Autónoma de Nuevo León (UANL), San Nicolás de los Garza 66455, México
[2] Centro de Investigación en Biotecnología y Nanotecnología, Facultad de Ciencias Químicas, Universidad Autónoma de Nuevo León, Parque de Investigación e Innovación Tecnológica, Apodaca 66628, México

* Correspondence: jose.moronesrmr@uanl.edu.mx.

Supplementary Material

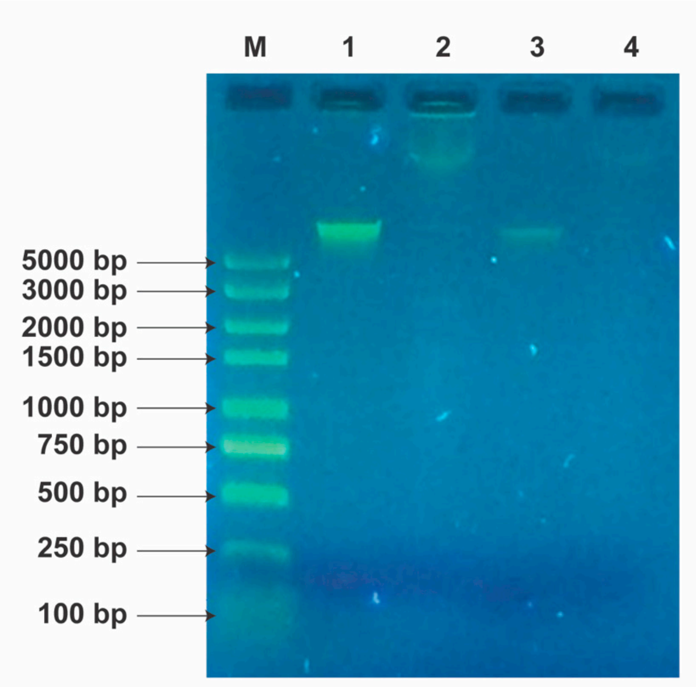

Figure S1. Viral DNA extraction from bacteriophages M8-2 and M8-3. Electrophoresis was performed using a 0.8% agarose gel in 1X TBE buffer. M: Molecular weight marker 100-5000 bp DNA Marker Plus (Bio line); Lane 1: Viral DNA from phage M8-2; Lane 3: Viral DNA from phage M8-3; Lanes 2 and 4 represent the cell lysate stock for M8-2 and M8-3, respectively.

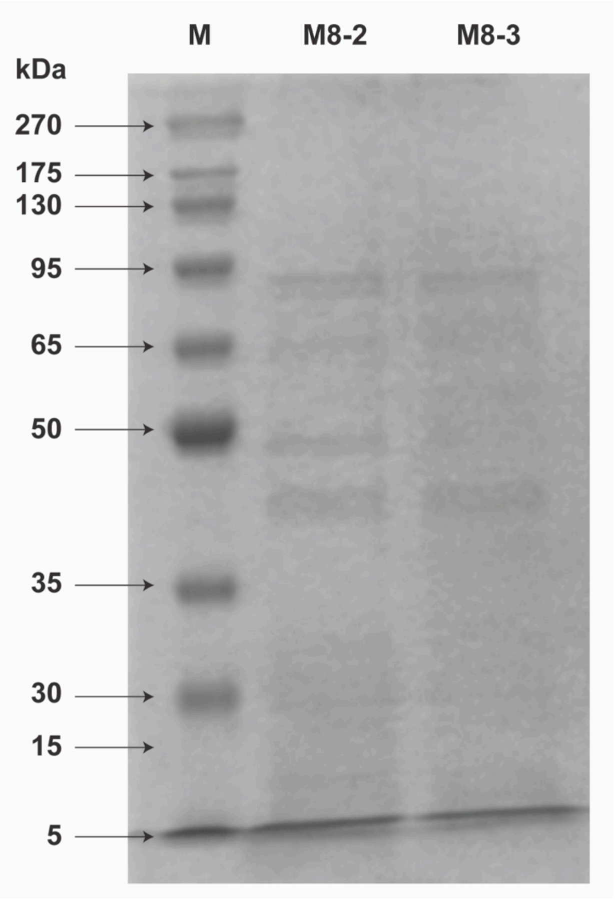

Figure S2. SDS-PAGE analysis of structural proteins in phages M8-2 and M8-3. Polyacrylamide gel electrophoresis was conducted using a concentrating gel (4%) and a separating gel (12%). M: Broad Multi Color Pre-Stained Protein Standard (GenScript); M8-2: Structural proteins of bacteriophage M8-2; M8-3: Structural proteins of bacteriophage M8-3. The banding patterns reveal five resolved proteins with approximate molecular weights of 95 kDa, 65 kDa, 50 kDa, 40–45 kDa, and 30 kDa.

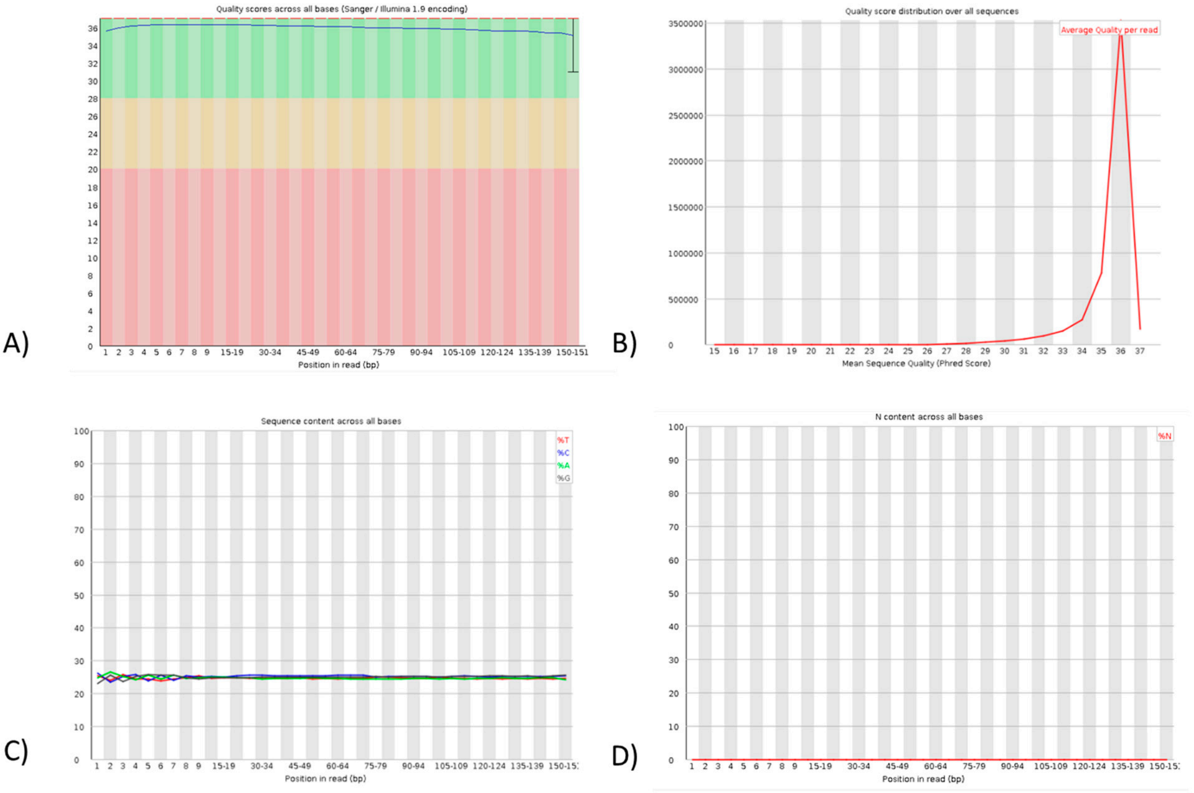

Figure S3. *Quality analysis with FastQC of the raw sequencing reads of phage M8-2.* (**A**) Average Phred scores (Q-values) across the sequence length (151 bp): The line within the green area represents the quality scores (y-axis) across the sequencing read length (*x*-axis). (**B**) Distribution of Q-values across all sequencing reads. (**C**) Nitrogenous base content across the genome: Four parallel lines indicate no discrepancies in base calling throughout the sequencing read. (**D**) Content of “N” assignments (any nucleotide) across the sequencing read length.

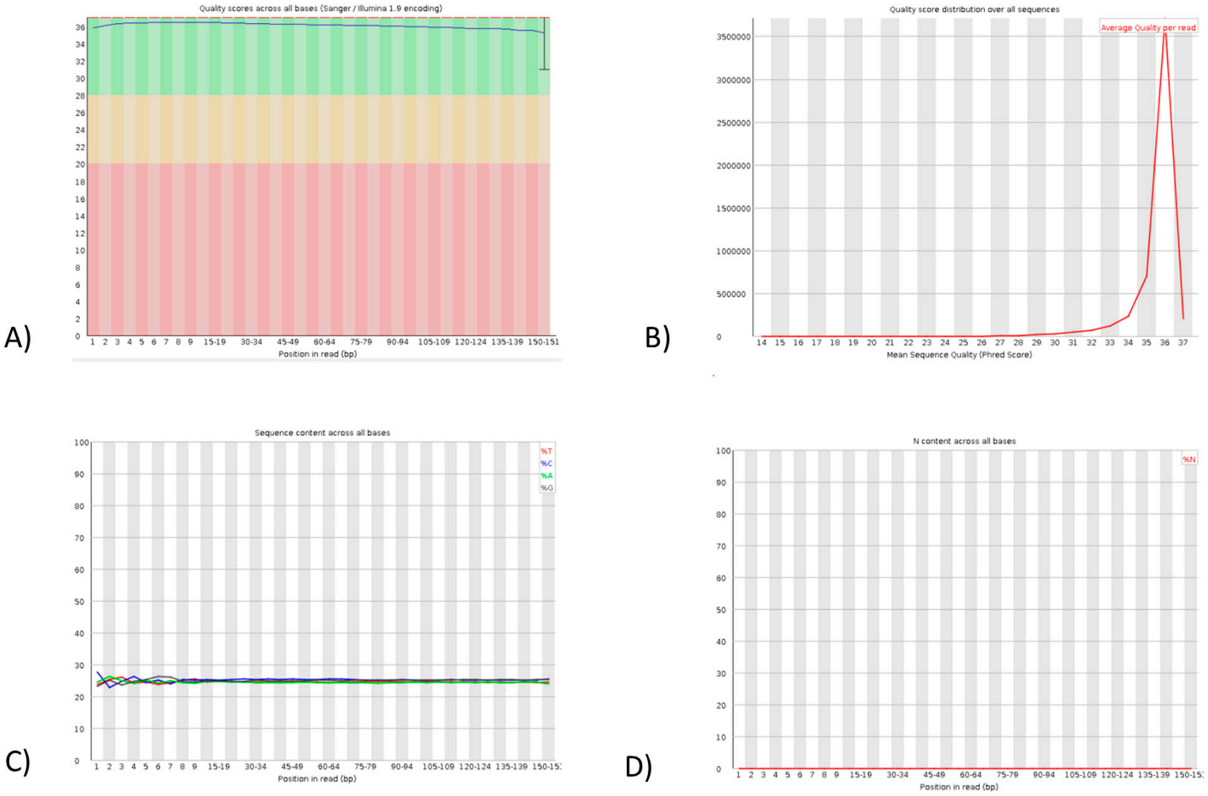

Figure S4. *Quality analysis with FastQC of the raw sequencing reads of phage M8-3.* (**A**) Average Phred scores (Q-values) across the sequence length (151 bp): The line within the green area represents the quality scores (y-axis) along the sequencing read length (*x*-axis). (**B**) Distribution of Q-values across all sequencing reads. (**C**) Nitrogenous base content across the genome: Four parallel lines indicate no discrepancies in base calling throughout the sequencing read. (**D**) Content of “N” assignments (any nucleotide) across the sequencing read length.

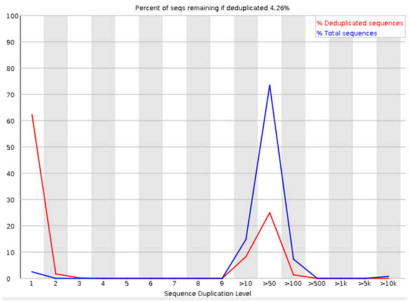

A)

Sequence	Count	Percentage	Possible Source
AGGGACACATAGAGATGTACTATCGAACTGGTACACTAGGACACTCTTTA	30301	0.5860216592645999	No Hit
TCTCACAGTTCAAGAACCTCAAGTACCCCCCCATAGCCCTCTTAAAGCC	14020	0.2711469477208571	No Hit
TCTCACAGTTCAAGAACCTCAAGTACCCCCCATAGCCCTCTTAAAGCCA	6672	0.1290365502991126	No Hit

B)

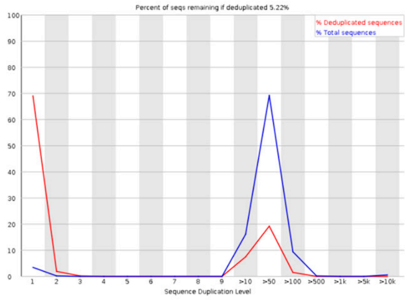

C)

Sequence	Count	Percentage	Possible Source
TCTCACAGTTCAAGAACCTCAAGTACCCCCCATAGCCCTCTTAAAGCCA	16239	0.31237706849539054	No Hit
AGGGACACATAGAGATGTACTATCGAACTGGTACACTAGGACACTCTTTA	15961	0.30702939776186516	No Hit
CTCACAGTTCAAGAACCTCAAGTACCCCCCATAGCCCTCTTAAAGCCAC	5928	0.11403234571344756	No Hit

D)

Figure S5. *Low-quality metrics by FastQC in the raw sequencing reads of phages M8-2 and M8-3.* (**A**) Percentage of duplicated reads in the raw sequencing data of phage M8-2. (**B**) Overrepresented sequences in the raw sequencing data of phage M8-2: Three highly repetitive sequences were found throughout the sequencing data for phage M8-2. (**C**) Percentage of duplicated reads in the raw sequencing data of phage M8-3. (**D**) Overrepresented sequences in the raw sequencing data of phage M8-3: Three highly repetitive sequences were found throughout the sequencing data for phage M8-3.
